# Socially engaged calves are more likely to be colonised by VTEC O157:H7 than individuals showing signs of poor welfare

**DOI:** 10.1038/s41598-020-63186-2

**Published:** 2020-04-14

**Authors:** Lena-Mari Tamminen, C. Reed Hranac, Johan Dicksved, Erik Eriksson, Ulf Emanuelson, Linda J. Keeling

**Affiliations:** 1 Department of Clinical Sciences, Swedish University of Agricultural Sciences, Box 7054, SE-75007 Uppsala, Sweden; 2 Molecular Epidemiology and Public Health Laboratory (mEpilab), Infectious Disease Research Centre, Hopkirk Research Institute, Massey University, Private Bag, 11-222 Palmerston North, New Zealand; 3 Department of Animal Nutrition and Management, Swedish University of Agricultural Sciences, Box 7024, SE-75007 Uppsala, Sweden; 4 National Veterinary Institute (SVA), SE-75189 Uppsala, Sweden; 5 Department of Animal Environment and Health, Swedish University of Agricultural Sciences, Box 7068, SE-75007 Uppsala, Sweden

**Keywords:** Applied microbiology, Agroecology

## Abstract

In cattle herds, the transmission and persistence of VTEC O157:H7 (a serotype of verotoxin-producing *Escherichia coli* – known for its life threatening complications in humans) is dependent on a small proportion of cattle who become colonised and shed high numbers of the bacteria. Reducing the proportion of these animals is considered key for decreasing the prevalence of VTEC O157:H7. In this study, observations of calf behaviour and animal-based welfare indicators were used to explore individual risk factors and underlying drivers of colonisation in Swedish dairy calves. Interdependencies between variables led to three different approaches being used to visualize and explore the associations. Combining the results of all methods revealed similar patterns and suggest that healthy animals, actively grooming and interacting with others calves in the group have a higher risk of colonisation than small dairy calves in poor condition (diarrhoea, poor ruminal fill, poor body condition score and nasal discharge). This lends no support to the hypothesis that reduced welfare is a risk factor for VTEC O157:H7, but implies that individual differences in calf behaviour affect oral exposure to the bacteria so driving the risk of colonisation. This new finding has important implications for understanding of VTEC O157:H7 transmission within farms.

## Introduction

The zoonotic pathogen verotoxin-producing *Escherichia coli* serotype O157:H7 (VTEC O157:H7) causes disease and even deaths among people worldwide and Sweden is over represented, having had several major outbreaks^1–3^. Many of the Swedish cases of VTEC O157:H7 are connected to strains circulating in the cattle population^2,4,5^ and there are higher numbers of human cases in cattle dense areas^6^. Although experimental infection with high doses of VTEC O157:H7 may cause diarrhoea in young calves^7^ it is does not appear to be generally associated with disease^8^ and the most significant animal reservoirs are cattle asymptomatically carrying VTEC O157:H7 in their intestines^9^. The primary site of colonisation is considered to be the recto-anal junction in the terminal rectum and colonisation of this region has been associated with high levels of bacterial shedding^10–12^. Shedding of more than 10^4^ colony forming units (cfu) per gram has been proposed to indicate a “super-shedder”^13^ and modelling has suggested that these individuals are responsible for shedding as much as 90% of total bacteria in a group^14^. It has also been shown that high-level shedders increase contamination levels in the environment, on the hide of animals and increase average shedding levels of pen mates^15–17^. More recent studies have questioned the focus on super-shedders and suggested that even lower shedding levels (>10^3^ cfu/g faeces) are important for on farm persistence and transmission^18^. In all cases, reducing the average shedding rate of animals is an important control measure for preventing transmission from cattle to humans^19,20^.

Although it appears that all calves can be colonised in experimental studies, using high inoculation doses^21,22^, on farm conditions present more complicated dynamics as transmission and pathogenesis of a bacteria is part of a complex interaction between host, bacteria and environment^23^. In field studies large variations in the prevalence of colonised animals has been observed and both experimental and field studies show considerable individual variation in the duration of colonisation and shedding levels^11,22,24–27^. This heterogeneity between individuals suggests that, in addition to bacterial presence, factors related to the host influence the colonisation process as reviewed by Munns *et al*.^28^. It is well documented that individuals differ in behaviour and in how well they are coping with their situation, and so they differ in their welfare^29–34^. However, the effect of individual differences in behaviour and welfare on colonisation of VTEC O157:H7 remain largely unexplored.

Research in the field of animal welfare has led to development and validation of animal-based indicators to assess welfare^35,36^. A novel approach in this cross-sectional study was to combine observations of animal-based welfare measures and behaviour to explore individual risk factors for colonisation in Swedish dairy calves exposed to VTEC O157:H7 and test the hypothesis that individuals with indicators of poor welfare would be more susceptible to colonisation. Three different analytical methods, with different strengths and weaknesses, were used due to the analytical challenges of having a large number of interdependent explanatory variables. These were cluster analyses, elastic net regression and principle component regression.

## Results and Discussion

A total of 318 calves were sampled across 12 farms positive for the environmental presence of VTEC O157:H7. Fifty-six calves were colonised as detected by recto-anal mucosal swabs (RAMS) from the terminal rectum and their faecal shedding levels varied between 0 and 840 000 cfu/g faeces (Table 1). Animal-based assessment of health and welfare of the 56 colonised individuals (cases) were compared to 135 non-colonised calves (controls) housed in the same pens (n = 26) to describe patterns in calf behaviour and welfare that may influence the risk of colonisation. In this way, we controlled for environmental and management factors associated with the pen. See Supplementary Table S1 for assessment-protocol.

**Table 1:** Pens where calves colonised by verotoxin-producing *Escherichia coli* serotype O157:H7 (VTEC O157:H7) were identified. IQR = Interquartile range.

Farm	Pen	Number of animals:		Mean age (days)	IQR age (days)	Proportion colonised* animals in pen	Number of shedders	Shedding levels^†^ (cfu/g)
		in pen	Sampled in pen					
1	1	5	4	94	9.5	100%	1	16000
	2	8	5	106	14	40%	0	—
2	1	11	8	145	28.8	13%	0	—
	2	12	5	196	24	20%	1	40
3	1	20	10	85	11.5	10%	1	700000
4	1	7	7	111	9.5	29%	1	66800
5	1	14	9	154	41	33%	1	143000
6	1	4	3	51	5.5	33%	1	45
	2	20	14	99	40.5	64%	0	—
7	1 (visit 1)	6	6	49	19.5	33%	0	—
	2 (visit 1)	4	4	95	23.8	75%	1	185000
	1 (visit 2)	7	7	78	30.5	43%	1	77272
	2 (visit 2)	7	7	121	35.5	14%	1	21318
8	1	18	8	219	15.8	13%	1	425000
	2	8	5	175	16	20%	0	—
	3	12	8	118	61.8	13%	0	—
9	1 (visit 1)	36	14	176	52.5	7%	1	360
	2 (visit 1)	8	5	98	1	40%	2	900;1300
	3 (visit 1)	10	5	101	11	40%	1	15100
	5 (visit 2)	8	7	101	12.5	43%	1	1360
10	1	12	12	61	29	17%	2	654000; 840000
	2	13	7	195	62	14%	0	—
11	1	4	4	71	6.3	75%	0	—
	2	6	6	171	28	33%	0	—
	3	3	3	43	25.5	33%	0	—
12	1	19	18	216	35.8	17%	2	300;600

*VTEC O157:H7 detected in recto anal mucosal swabs.

^†^Quantified presence of VTEC O157:H7 from faecal sample.

The number of colonised animals (RAMS positive) varied between the pens (range 0–9 individuals per pen) and only 19 of the colonised animals were shedding VTEC O157:H7 in their faeces (positive faecal sample). However, most of the shedding individuals shed high levels of bacteria (68% shed >10^4^ cfu/g, 89% shed >10^3^ cfu/g). PCR confirmation of two individual isolates from each farm showed that all farms except one had isolates with the virulence genes *eae* and *vtx2* (the exception had *eae* and *vtx1*). All isolates except the *vtx1* positive isolate came from farms located in a region known to have a high prevalence of a virulent strain of clade 8 and were likely closely related^37^. The remaining isolate was from a farm that was identified in association with an epidemiological investigation following a human case. Thus, all isolates in the study have the potential to cause disease when transmitted to humans.

### Visualising associations between animal-based and behavioural variables using cluster analysis

**Figure 1:**
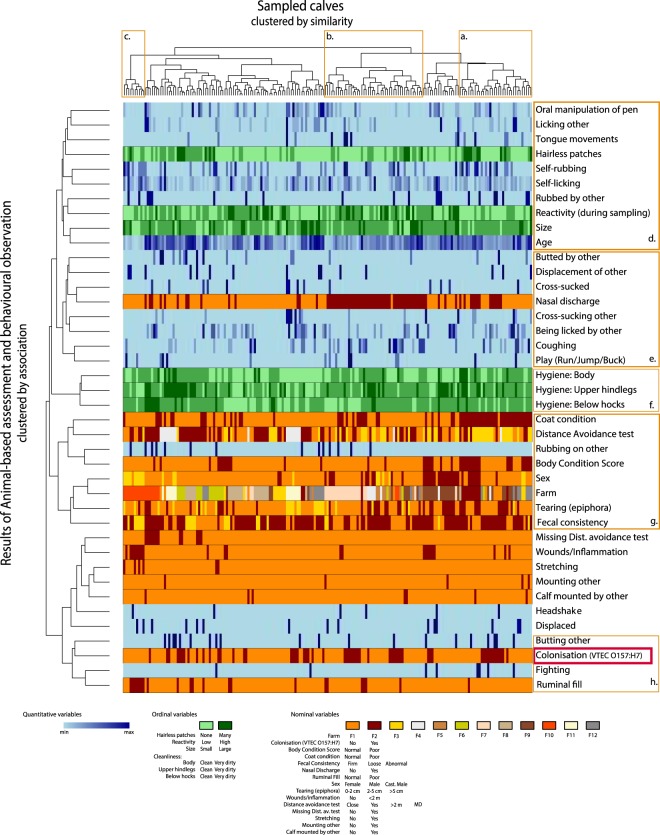
Cluster analysis including welfare and behavioural observations of the 56 colonised (positive recto-anal-mucosal swabs) and 135 non-colonised dairy calves from 12 Swedish farms infected with verotoxin-producing *Escherichia coli* serotype O157:H7 (VTEC O157:H7). The top horizontal axis represents each individual calf (clustered by similarity, using Gowers distance) and the animal-based variables (clustered by association) are presented on the left vertical axis. Results of the animal-based assessment of each variable (and which farm the individual belonged to) are illustrated by a vertical line below each individual. To aid references to this figure in the text, clusters are given the letters a-h.

The animal-based assessments and colonisation status of the sampled animals are visualised in Fig. 1 (more detailed results are found in Supplementary Table S2). The top horizontal axis of Fig. 1 represents each individual calf (clustered by similarity, using Gowers distance) and the animal-based variables (clustered by association) are presented on the left vertical axis. Noteworthy is that the colonised animals did not form a distinct individual cluster i.e. the swab positive calves were distributed along the horizontal axis and not to specific clusters of individuals (see red box. Fig. 1). Instead, individual similarity appeared to be based on many different factors. Calves with poor coat condition appeared to cluster (see cluster a. Fig. 1) as did calves with nasal discharge (see cluster b. Fig. 1). In addition, a cluster including calves, characterised by having wounds/inflammation and poor ruminal fill was identified (see cluster c. Fig. 1). This cluster also included a high proportion of the castrated males sampled within the study.

Associations between the animal-based assessments included a clustering of comfort behaviours like self-licking and rubbing. These behaviours clustered close to variables associated with oral manipulation of peers and the environment and having hairless patches as well as age, calf size, reactivity during sampling and being rubbed by other calves (see cluster d. Fig. 1). There was also a cluster which included receiving and performing active/social behaviours like being butted and displacing others, as well as playing, nasal discharge and coughing (see cluster e. Fig. 1). Hygiene related variables; such as cleanliness of lower hind legs, hind and body, formed a distinct cluster (see cluster f. Fig. 1). Farm clustered with a number of potentially management related variables like coat condition, fearfulness as indicated by the distance avoidance test, body condition score etc. (see cluster g. Fig. 1).

Variables clustering closely to colonisation status were butting of other calves, fighting and ruminal fill (see cluster h. Figure 1). Butting others appeared to be positively associated while poor ruminal fill appeared inversely related to colonisation. This is surprising, as dietary stress has previously been correlated with increased shedding post inoculation of a large number of VTEC O157:H7^38^. This may indicate that the effect of fasting is different for colonisation compared to shedding. The association with fighting is difficult to assess due to the low frequency of calves performing fighting behaviour (n = 8).

### Identifying predictors of colonisation status using elastic net regression

**Figure 2:**
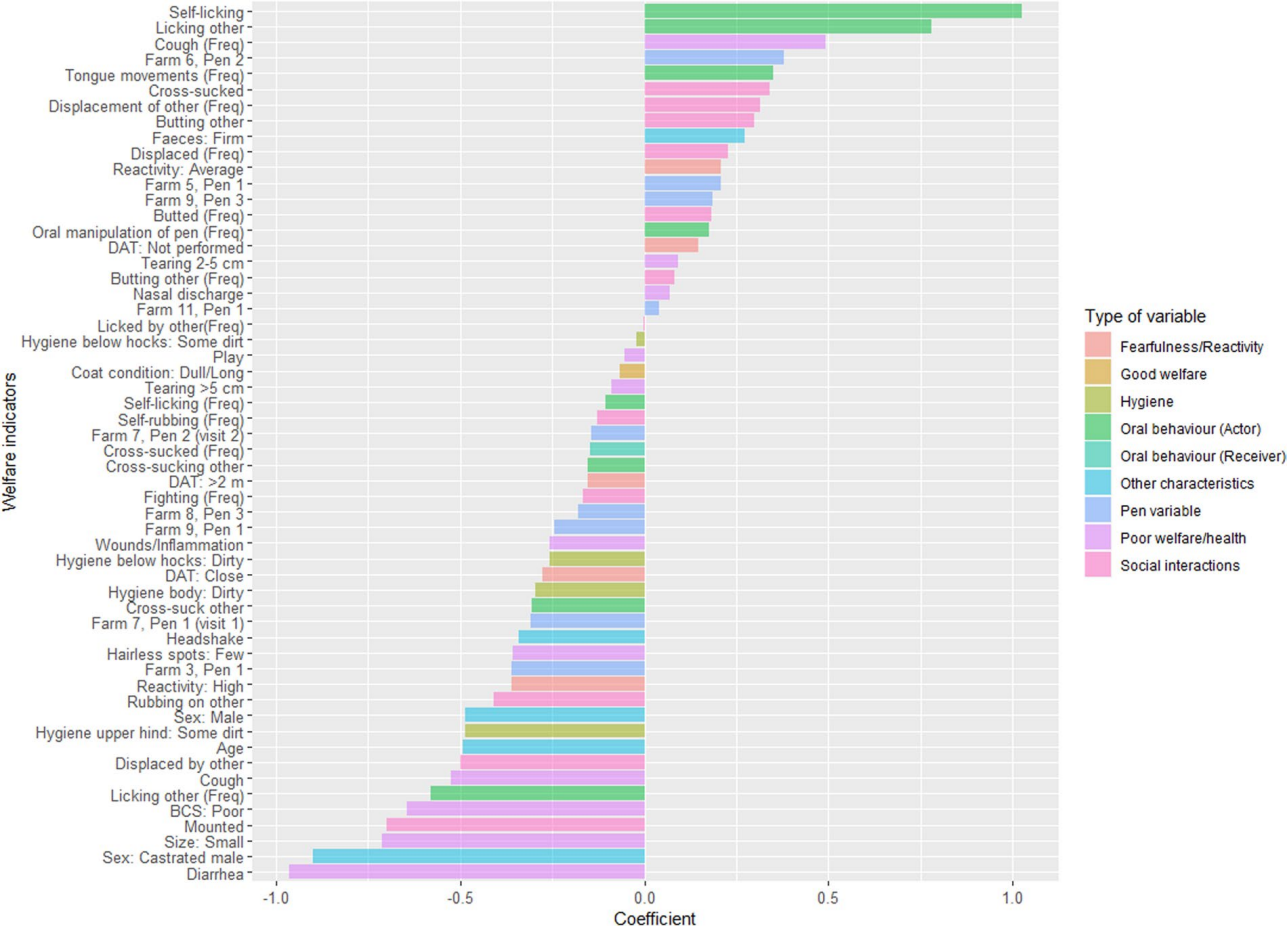
Coefficients in the elastic net regression model that maximised area under the curve (69.3%). Positive coefficients were positively associated with colonisation by verotoxin-producing Escherichia coli serotype O157:H7 and negative coefficients were negatively associated with colonisation. Behavioural variables marked with (Freq) are quantitative measures of the number of times the behaviour was observed as opposed to binary variables indicating whether a behaviour was observed (yes/no). The variables are grouped to help illustrate how the different types of variables are associated with colonisation.

Results from the behavioural and welfare assessments of the individuals, and pen-ID, were included in an elastic-net logistic regression model with colonisation (yes/no) as response variable. Coefficients for maximal area under the curve (AUC) after 10-fold cross-validation are presented in Fig. 2. Pen-ID was included to account for the fact that calves were kept in groups and for physical characteristics of pen. The identified individual variables are therefore relevant for all calves in any pen. The inclusion of pen-IDs in the final model indicates that, in addition to individual calf differences, there are pen level characteristics (social or physical) that influence colonisation risk. Understanding such characteristics may be important for reducing the prevalence of VTEC O157:H7 and should be considered in future studies.

The associations identified by the elastic net regression model were partly consistent with the cluster analysis. For example, butting others was positively associated with colonisation while mounting others and being displaced by others were negatively associated with colonisation. The elastic net regression also indicated positive associations between colonisation and oral behaviours, like self-licking and licking others. Performing these behaviours will of course increase exposure to the bacteria. Negative associations were observed with being small, castrated, having a poor body condition score and diarrhoea (firm faeces was positively associated with colonisation). This is consistent with a previous study observing a cohort of heifers on pasture where firm faecal consistency and high body condition score was associated with shedding of VTEC O157:H7^39^.

To handle the high frequency of zero observations in some of the behavioural variables, binary variables describing if an individual calf performed a behaviour (yes/no) were created and analysed alongside the quantitative variable of the number of times the behaviour was observed^40^. There were several examples where the binary variable had an opposite relationship with the outcome than the corresponding quantitative variable. For example, being a calf observed to perform self-licking and licking other calves was positively associated with colonisation while the number of observations of licking were negatively associated with colonisation. This indicates a difference between calves performing these behaviours at lower frequency compared to calves performing them at high rate, although this needs to be confirmed since the negative coefficient for self-licking frequency was relatively small.

Similarly, coughing, being displaced and displacing others also had opposite relationship between categorical and quantitative variables. Being observed to perform these behaviours was negatively associated with colonisation, but within the group of animals, performing the behaviour more often increased the risk of colonisation. For coughing there are multiple reasons for increased frequency of coughing. The most obvious is a more severe respiratory infection leading to coughing, but during observations calves often coughed in association with playful activities. This association is also evident in the cluster analysis where coughing and play were together in the same cluster. Thus, frequent coughing may be a poor indicator of respiratory disease as the higher frequencies are very influenced by a small number of playing individuals. The association between colonisation and increased frequency of displacements could be differentiating between calves that are displaced once, and then do not engage in further interaction, and calves that are actively participating in competition and social interaction with their pen-mates. Indeed, the overall picture when looking at all coefficients, both positive and negative, indicates that the colonised calves are not found among the sick, stressed and so potentially immunocompromised calves in a group, as hypothesized, but among the animals that are active, grooming and socially engaged. This is in keeping with the previous analysis showing that colonised calves did not cluster with those showing symptoms of sickness or stress, but close to those butting other calves, fighting and having good ruminal fill.

### Exploring interdependence and covariation using principal component regression

**Figure 3:**
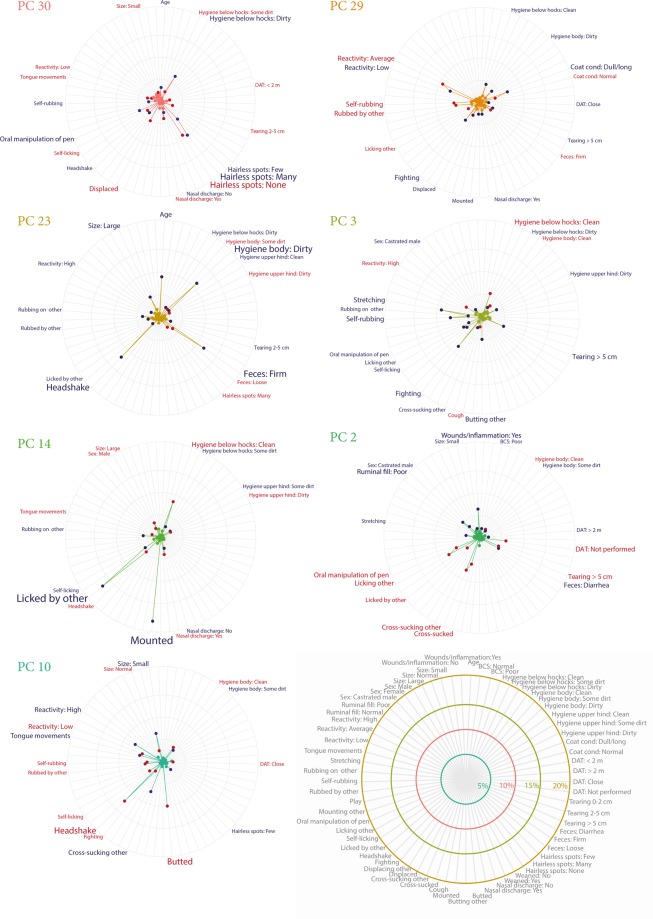
Variable contributions (%) for the seven significant principal components included in the final principal component logistic regression model. Variable contribution indicates how much influence the individual variables have within each principle component - the longer the line (i.e. the greater the %) the more influential that particular variable. Red variables are positively associated with colonisation of verotoxin-producing *Escherichia coli* serotype O157:H7 and blue variables negatively associated. Each principle component represents a different aspect of the variation in the whole dataset, so the variables within each component should be interpreted together. DAT = Distance avoidance test.

The 39 variables from the animal-based assessment were combined using principal component analysis (PCA). Thirty-two principal components (PC) with eigenvalue > 0.6 (explaining 87% of the variation in the data, each component between 5.7–1.3%) were subsequently included in a logistic regression. After backwards stepwise selection using Akaike information criterion (AIC) the final PC regression model included 14 PCs associated with colonisation of which 7 had a p-value < 0.05. Estimates from the logistic regression are presented in Table 2. The variable contributions (in percent) to the significant PCs are presented in Fig. 3. Contributions of all components included in the model can be found in Supplementary Fig. S3.

**Table 2:** Components included in the final logistic regression model using principal component scores to predict colonisation of verotoxin-producing Escherichia coli serotype O157:H7 (sorted by p-values). AUC = 81.2%. DAT = Distance avoidance test.

Principal Component information:				Principal Component regression:		
*PC*	*Eigenvalue*	*Variance explained*	*5 most influencing variables: (Contribution to component)*	*b*	*SE*	*p-value*
30	0,69	1,41	Hairless patches: Many (10%), Hairless patches: None (9%), Hygiene below hocks: Dirty (7%), Hairless patches: Few (6%), Oral manipulation of pen (5%)	−0,72	0,25	0,004
29	0,73	1,49	Reactivity: Average (9%), Reactivity: Low (7%), Coat condition: Poor (7%), Self-rubbing (6%), Rubbed by other (5%)	−0,57	0,23	0,014
23	0,90	1,83	Headshake (12%), Faeces: Firm (12%), Hygiene body: Dirty (11%), Age (9%), Size: Large (5%)	−0,45	0,20	0,025
3	2,32	4,73	Stretching (9%), Fighting (8%), Self-rubbing (7%), Hygiene below hocks: Clean (5%), Tearing >5 cm (5%)	−0,35	0,17	0,036
14	1,28	2,60	Mounted (19%), Licked by other (17%), Hygiene below hocks: Clean (8%), Headshake (5%), Self-licking (4%)	−0,38	0,18	0,036
2	2,70	5,51	Cross-sucking other (8%), Licking other (8%), Wounds/Inflammation: Yes (6%), Cross-sucked (6%), DAT: Not performed (6%)	0,27	0,13	0,038
10	1,50	3,06	Headshake (12%), Butted (10%), Reactivity: High (7%), Cross-sucking other (7%), Size: Small (7%)	0,32	0,16	0,044
17	1,13	2,30	Fighting (10%), DAT: > 2 m (7%), Displacing other (6%), Oral manipulation of pen (6%), Sex: Male (5%)	−0,38	0,20	0,057
6	1,83	3,74	Butted (10%), DAT: Not performed: (7%), Licking other (7%), Tongue movements (7%), Hairless spots: Few (6%)	−0,29	0,15	0,058
8	1,57	3,21	DAT: Close (11%), Self-licking (9%), Displacing other (6%), Rubbed by other (5%), Oral manipulation of pen (4%)	0,30	0,17	0,071
28	0,76	1,55	Cross-sucked (15%), Fighting (7%), Faeces: Abnormal (6%), Oral manipulation of pen (6%), Mounted (5%)	−0,39	0,23	0,091
4	2,09	4,27	Play (8%), DAT: < 2 m (6%), Self-scratching (6%), Tongue movements (6%), Age (4%)	0,23	0,14	0,098
20	1,04	2,13	Reactivity: High (10%), Mounting other (9%), Displaced (9%), Mounted (6%), Play (5%)	−0,34	0,21	0,107
11	1,43	2,92	Size: Small (14%), Rubbing on other (8%), Displacing other (8%), Rubbed by other (5%), Hygiene upper hind: Clean (5%)	−0,27	0,17	0,113

The importance of several associations suggested by the elastic net analysis are confirmed in the significant components of the PC regression. The association between social behaviours and poor welfare are especially clear within PC 2 (p = 0.038) (Fig. 3) where oral manipulation of the environment as well as performing and receiving grooming (licking) and cross-sucking was positively associated with colonisation. In contrast indicators of poor welfare (like wounds/inflammation and poor ruminal fill) were negatively associated with colonisation. Interestingly this PC indicates a correlation between the social and oral behaviours and that the distance avoidance test (DAT) not being performed (due to restricted space) which may indicate that these types of behaviours are occurring more frequently in smaller, more densely populated pens. Self-licking, which received the largest coefficient in the elastic net regression, appeared in several of the significant PCs but was not among the most influential contributors on any except for the non-significant PC 8 (p = 0.071) where self-licking was the second largest contributor (Table 2). This component also indicated a correlation between self-licking, rubbing, oral manipulation of pen and displacing others and that these were linked with colonisation (Supplementary Figure S3).

Other PCs that revealed a pattern similar to PC 2 (i.e. appeared to separate calves with good health and welfare from calves that were coping less well) were PC 30 (p = 0.004) and 29 (p < 0.014). PC 30 differentiated between calves without hairless spots and calves that were dirty below the hocks and had few or many hairless spots. PC 29 separated calves with a normal coat and average reactivity, performing self-rubbing, from calves with a poor coat condition and low reactivity. Looking more closely at these components, they also visualise the complex interdependencies within the data set. For example, in PC 30 being displaced and having low reactivity were positively associated with colonisation but on PC 29 they were negatively associated with colonisation. This suggests that these variables have context dependent relationships. This may relate to the finding that calves displaced more frequently were more at risk for colonisation compared to calves displaced at lower frequency observed in the elastic net regression.

Component 23 (p = 0.025) is most likely describing a protective effect of increasing age which has been previously observed^41^. It also highlights a correlation between increasing age, dirtiness and firm faeces. This association may explain why poor hygiene appears to be negatively associated with colonisation on multiple components. However, considering the cross-sectional design of this study it could also be that calves kept in a dirty environment, with high infection pressure, actually may have been previously colonised and therefore less likely to be colonised again due to immunity.

Being a male or a castrated male was associated with reduced risk of colonisation in the elastic net regression. While some previous studies have identified castrated males as less likely to shed VTEC O157:H7^42^ and that heifers and cows are more likely to be colonised^17^, others have identified very small differences between sexes^41^. In the PC regression, being a castrated male was associated with reduced risk in PCs 2 and 3 and being a non-castrated male was associated with increased risk in PC14. Due to the small number of observed castrated male calves (n = 19) it is likely that the associations observed are a result of correlations between variables and thus not a direct effect of sex on colonisation risk. Similarly, PC 14 is highly influenced by being mounted by others. This variable was picked up by the elastic net regression as well as by the cluster analysis but as it was observed at very low frequency (n = 7) there is also a risk for spurious associations.

#### General discussion

The aim of this study was to investigate the association between welfare and colonisation of VTEC O157:H7 as well as explore patterns of behaviour and welfare to identify risk behaviours. The main hypothesis was that calves with indicators of poor welfare are more likely to be experiencing stress and stress-induced immunosuppression and therefore more susceptible to colonisation compared to other individuals in the same pen^43,44^. However, several variables traditionally used to define poor welfare were negatively associated with colonisation. This was especially clear in the results of the elastic net regression where, for example, having diarrhoea, being displaced and having poor body condition score were negatively associated with colonisation. This was supported by the principal component regression where similar patterns were observed within several significant components. Thus, there was no support for the suggestion that individuals with poor welfare are more likely to become colonised. In fact, the results seem to support the opposite view, that it is active, social individuals (involved in agonistic interactions and possibly high exertion play movements) who are colonised.

There were indications that colonised animals may be more frequently exposed to the bacteria by performing more comfort behaviours (i.e. self-licking) and by grooming others. Self-licking is a behaviour with many motivators^45^ but one of the most obvious drivers is a direct response to being dirty, meaning that the calves that are in a dirty environment and are licking to clean themselves are more likely to ingest the bacteria. These calves would be cleaner which could explain why hygiene is not emerging as a risk factor in our analysis. In addition, grooming and cleaning behaviours require an energy investment and it is for this reason that sick individuals do not groom themselves as often^46^. This could explain the association between non-colonised animals and poor health and welfare observed in both the elastic net regression and PC regression.

Social grooming, besides also increasing exposure to the bacteria, plays an important role for tension relief as well as maintaining and building relationships^46^. The observed connection between colonisation and calves performing social grooming and agnostic behaviours are interesting from a disease spreading perspective. The distinction between super-shedders (individuals shedding high levels of bacteria) and super-spreaders (individuals with more contacts/interactions with other hosts) as independent traits has previously been introduced for VTEC O157:H7^13^. Results of this study are the first to provide evidence that risk factors for colonisation are also associated with behaviours related to social contacts and interactions with peers, i.e. risk behaviours for super-spreading.

From the PC regression, it is clear that there are complex interdependencies among the predictors that jointly influence the colonisation process. A common method for handling many variables is reducing the data by removing some of the variables based on biological reasoning and the focus of the research question. However, when prior information is lacking the decision of which variables to remove can be difficult and lead to oversimplification and bias. In addition, the possibility to consider associations between variables, which may provide important information for interpretation of results, disappears. In this study we approached this by combining multiple methods with different strengths and weaknesses. Combining the results of all methods revealed similar patterns and the methods strengthen each other as well as reveal different aspects influencing the analysis.

As a first step we used cluster analysis to visualise associations and data. This provided a good overview of variables and indications of correlation and associations. However, there was no way to assess goodness of fit and clusters may change drastically if variables are excluded. The elastic net regression enabled analysis of all variables (including the 26 pens as fixed effects to control for non-independence within pens) and provided a reduced model that still included correlated variables, like self-licking and licking others, even if this induced variance inflation when combined together in a logistic regression. However, the limitation of this approach remains that the coefficients generated are biased estimates and cannot be used to calculate odds ratios to quantify the importance of the predictors. In addition, the area under the curve (AUC) for the best model presented in this paper was 69.3%. This means that the individual variables only gave us part of the picture. The AUC for the PC regression, on the other hand, was 81.2%, indicating that the inclusion of non-independent data increased model performance and was better at capturing the complex interdependencies of welfare metrics. In addition, it exposed interesting correlations between variables, deepened the understanding of associations and revealed potential sources of bias in the study. However, the limitation of the PC regression method is that the creation of components is driven by variation within the data and therefore unrelated to outcome. There may be variables that are strong predictors for colonisation, but if they do not explain variation within the data they will not be represented on any component and so will not be identified. Another drawback of this method is that it can be difficult to infer the biological pattern represented by each components. The strength of using these different approaches is the increased confidence in associations identified within all three methodologies. Nevertheless, it should be noted that some associations may have been missed due to the relatively short behaviour observation period or the number of animals (i.e. there is a risk of type II errors).

Although the cross-sectional study design means that causality cannot be inferred we have managed to tease apart the complex structure of individual patterns. We suggest that future studies test the hypothesis that performing behaviours related to oral exposure, like self-licking and social grooming, increase the risk of colonisation in calves. In addition, the underlying association between poor welfare indicators and reduced risk of colonisation should be further investigated to identify if this is due to immunity from earlier infection (with this or similar strains of bacteria), decreased exposure (e.g. due to sickness behaviour) or differences in colonisation resistance (e.g. variations in the microbiome or other features of the terminal rectum of these individuals). As others before^47^, we suggest longitudinal studies with increased frequency of observations that will enable the investigation of how individual colonisation resistance/susceptibility changes over time in relation to changes in behaviour, previous infection and increasing age. The individual drivers of colonisation emerging in this study have important implications for understanding of VTEC O157:H7 transmission within farms as well as for developing on-farm measures for reducing the prevalence of the bacteria on farms in the future.

## Conclusion

The results of this study suggest that small dairy calves in poor condition (diarrhoea, poor ruminal fill, poor body condition score and nasal discharge) are associated with a reduced risk of colonisation of VTEC O157:H7 compared to healthy animals that are actively grooming and interacting with others. This suggest that oral exposure to the bacteria is driving risk of colonisation in groups of calves.

## Materials and Methods

### Study population

In the study 318 animals, between 7 and 306 days of age, from 12 Swedish dairy farms with VTEC O157:H7 were sampled. Farms were visited between October 2015 and April 2017 and two of the farms were visited twice with a year between the visits. The farms were conventional farms with between 90 to 600 animals on farms. The majority of the farms had free-stall barns with milking parlour or automatic milking systems. One farm had a tie-stall system. Calves were generally kept in single crates for the first 2 weeks and then group housed. Weaning occurred around 2 months of age. Sampling and handling of animals was carried out in accordance with the ethical approval granted by the regional ethical committee (Uppsala Djurförsöksetiska Nämnd, Dnr: C 85/15). All methods were carried out in accordance with relevant guidelines and regulations.

### Identification of farms with VTEC O157:H7

The positive farms were identified based on results from a targeted environmental sampling of 59 farms in areas with suspected high prevalence of virulent VTEC O157:H7 (Öland, Skåne, Blekinge, Falkenberg). One farm was recruited in association with an outbreak of human disease from another area (Småland). Environmental sampling was performed by the first author or personnel from the local livestock association in accordance with national guidelines for investigating a farm suspected to be associated with an outbreak of VTEC O157:H7. This included collecting samples from calves (2–6 months of age) and young stock (6–12 months). From each age group a so-called overshoe (OS) sample was collected by fitting a gauze soaked with phosphate buffer over plastic overshoes. The gauze was rotated as the sampler walked around the pens. While walking around the pens a pooled faecal (PF) sample consisting of fresh manure from 15––20 pick points around the pen was collected. Samples were pre-enriched in tryptic soy broth (supplemented with 20 mg/l of novobiocin) at 41.5 °C ± 0.5 °C for 18–24 h before immunomagentic separation (IMS) (Dynabeads anti-E. coli O157; Thermo Fisher). Paramagnetic beads were spread on sorbitol McConkey agar supplemented with potassium tellurite (2.5 mg/l) and cefixime (0.05 mg/l) (CT SMAC) and incubated (37 °C for 18–24 h). Up to 5 suspected colonies were picked for agglutination with a latex kit (DR 622; Oxoid) and if positive for VTEC O157:H7 genes encoding O157 (*rfbO157*), verotoxin 1 *(vtx1)* and verotoxin 2 *(vtx2)* as well as intimin (*eaeA)* were established by PCR^48,49^. In total 14 positive farms were identified but one declined further participation. In addition, one farm was later excluded as no positive individuals were identified in the individual sampling leading to the final 12 farms included in the study. Analysis of isolates by real-time PCR (as described by Söderlund *et al*.^50^) established that 11 of the farms had the highly virulent type clade 8. The remaining farm was the one associated with a human outbreak of VTEC O157:H7 (*eae* and *vtx1* positive).

### Identification and sampling of pens with animals shedding VTEC O157:H7

To avoid unnecessary and time-consuming individual sampling, a more thorough environmental sampling to identify groups of animals shedding VTEC O157:H7 was performed on the positive farms. The first author or personnel from the local livestock association collected OS samples from non-weaned calves (approx. 0–2 months), weaned calves (2–6 months) and young stock (6–12 months). If the groups consisted of more than fifty animals in multiple pens, the pens were grouped and separate OS samples taken so that no OS sample represented more than 50 animals. If animals within the described age groups were housed in several buildings, separate environmental samples were collected from each building. Analysis of samples was performed as described above. Farms were revisited within two weeks of the thorough environmental sampling and animals housed in pens where VTEC O157:H7 had been isolated were included in the individual sampling. The aim was to sample a minimum of 20 calves from positive pens on each farm but on farms with many positive pens the number was increased to cover all pens and increase likelihood of identifying colonised individuals. However, the practical limit was around 30 individuals per farm. When all calves could not be sampled, selection of animals within positive pens was done by systematic randomisation (selecting the calf closest to the observer and then every second or every third calf by distance to the observer) or by lottery. Only individuals from pens where VTEC O157:H7 positive animals were identified were included in the final analysis. More information about proportion of sampled animals within pens and type of pens can be found in supplementary table S4.

### Individual health and welfare assessment of animals

Farm visits started when farmers began working in the barns in the morning (between 5:00 and 7:00 AM) and began with behavioural observations of a pen of undisturbed animals. These observations were carried out close to feeding time as this has been shown to be an active period^51^. Each pen where VTEC O157:H7 had been isolated was observed for 20 minutes on one day. The observer (the first author) was standing still outside the pen, at a minimum of 1 m from the pen. On farms where several pens were included, the observer rotated to the next pen every fifth minute so pens were observed four times. The behaviours observed included social and agonistic behaviours (like displacement, fighting, mounting other calves), comfort behaviours (like licking itself, scratching itself), stereotypies, disease related behaviours (coughing, lameness) as well as play. After undisturbed observation the observer entered the pen and assessed fearfulness by performing an avoidance distance test (DAT). A freestanding calf was approached from approximately 3 meters and at one arms distance the observer stopped and lifted her arm to touch the animal. The distance at which avoidance reaction occurred was noted. When pens were too small to be able to start the test from more than 2 meters the test was not performed. Each animal was then individually assessed using a selection of animal-based parameters including ruminal fill, body condition score, coat condition, cleanliness, signs of disease (cough, nasal discharge and diarrhoea) and wounds/inflammation. In addition the animal’s reactivity during sampling was scored. All behavioural and clinical observations were performed by the first author (a veterinarian) and the protocol was developed and practiced together with an ethologist (last author). After practice, it was piloted on 2 farms where some calves were assessed two times to confirm consistency. More details of the scoring protocol for the individual assessment can be found in the supplementary material (Supplementary Table S1).

### Sampling and microbiological analysis of VTEC O157: H7

Faecal samples from each animal were obtained from the rectum and placed in plastic jars. The recto anal junction of each animal was then swabbed using a foam coated cotton swab during a minimum of 1 minute. Sampling was performed by the first author and a new and clean pair of gloves were worn for each individual sampling. The recto anal mucosal swab (RAMS) was put into a 15 ml sterile tube containing 2.5 ml sterile phosphate buffer. The swabs were taken straight from manufacturers packaging before sampling and handled as sterile to avoid faecal cross-contamination. Packages were stored so they could not be exposed to VTEC O157:H7. Samples were sent to the Swedish national veterinary institute (SVA) in Uppsala for analysis and processed within 2 days following sampling.

Upon arrival at SVA, faecal samples were stored in 2°C while all swabs were analysed for VTEC O157:H7. RAMS were vortexed and 20 ml of modified tryptic soy broth (mTSB) (supplemented with 20 mg/l of novobiocin) was added before pre enrichment at 41.5° ± 0.5° for 18–24 hours, IMS and culture on CT SMAC followed by screening of sorbitolnegative colonies by agglutination as described above. Calves were considered colonized if *E. coli* O157 was isolated from RAMS and confirmed as O157 by agglutination. From each farm two isolates of *E. coli* O157 were chosen for additional confirmation by PCR and to identify the presence of genes coding for *rfbO157*, *vtx1* and *vtx2* and *eaeA*^48,49^.

The faecal samples of RAMS positive calves were analysed to quantify shedding levels. Ten grams of faeces and 90 ml of peptone salt solution were homogenised in a stomacher followed by a series of 10-fold dilutions. From each dilution, 0.1 ml was spread on CT SMAC agar. After incubation in 37°C for 18–24 hours, the number of sorbitol negative colonies were counted to quantify shedding levels and from this number the concentration of bacteria per g of faeces was calculated.

### Data management and statistical analysis

Data was entered in Excel and all statistical analysis was performed in R^52^. Figures were prepared using ggplot2^53^ and Adobe Illustrator^54^. All behavioural observations and age were treated as numerical variables, which were scaled and centred before analysis. Of the 318 observed calves, 44 calves had missing data, which were imputed using nonparametric random forest imputation in the package missForest^55,56^. Missing data occurred indiscriminately due to unreadable notes, in particular for the clinical observations where the evaluator called out the observation to another person keeping manual records outside the pen. The exception was the variable DAT, which had the highest proportion of missing observations (5%), which was handled separately as the missing individuals could represent high avoidance calves (thus missing systematically), by adding an additional categorical variable including missing DAT. The variable DAT also contained 12 observations where data was missing by design, as calves were housed in pens that were too small for the test to be performed. These observations were not imputed but handled as a separate category (DAT not performed). The proportion of missing observations in the remaining variables was less than 1% for 9 variables and less than 5% for 6 variables.

In the statistical analysis, RAMS-positive individuals (cases) were compared only to calves housed in the same pens with negative RAMS (controls) to avoid including animals that had not been exposed to the bacteria. This allowed us to investigate individual differences between colonised and non-colonised animals within groups exposed to similar external risk factors (for example same pen characteristics, management, feed). Thus, of the 318 observed calves, 191 were included in the analysis. To visualize the data, a hierarchical cluster analysis of variables was performed using the package CluMix^57^. Calves were clustered by similarity using Gowers distance and variables clustered using a combination of association measures according to the CluMix-ama approach^58^. The combined effects of each individual risk factors and their association with colonisation of VTEC O157:H7 were analysed using Elastic net regression in the package glmnet (alpha = 0.5)^59^. As many behavioural observations included a high number of calves that did not perform the behaviour, leading to a distribution with a high frequency of zero observations, a categorical variable describing if the behaviour was observed (or not) and a quantitative variable with the number of times the behaviour was observed was included^40^. Those behaviours which were only observed once per calf (mounted by other, mounting other and stretching), were only included as qualitative variables to avoid unnecessary collinearity. The model was fitted with 10-fold cross validation and the final model was chosen based on the lambda that generated the largest area under the curve^60,61^. Pen ID was included as a fixed effect to account for confounding on pen-level. Finally, a multivariate approach, performed by using the package PCAmixdata^62^ was used to generate non-correlated principal components (PCs) representing variation within the predictors. This was followed by generalized logistic regression where the generated PCs were used to predict colonisation status using the “stats” package^52^ and the package lme4^63^. All PCs with eigenvalue > 0.6 (n = 32) were included in the regression. Pen was not included when creating the components, but it was included as a random variable in the regression analysis to account for non-independence within pens). As Pen ID explained very little variation, it was excluded and the model reduced by stepwise, backwards selection based on the Akaike Information Criteria (AIC). After model reduction, pen was again included as a random variable and still explained little variance and did not change coefficients of the included components. Each excluded component was reintroduced in the final model. No component reduced AIC nor changed the effects of the final components more than 20%. For interpretation of patterns, the variable coefficients of significant components were used^64^.

## Supplementary information

Supplementary information.

## Data Availability

The datasets generated and analysed during the study are available in the Mendeley Data repository, 10.17632/nrkkp4sbc9.1.

## References

[1] Sartz L (2008). An outbreak of *Escherichia coli* O157:H7 infection in southern Sweden associated with consumption of fermented sausage; aspects of sausage production that increase the risk of contamination. Epidemiol. Infect..

[2] Folkhälsomyndigheten. Enterohemorragisk *E. coli* infektion (EHEC). (2019). Available at: http://www.folkhalsomyndigheten.se/amnesomraden/statistik-ochundersokningar/sjukdomsstatistik/enterohemorragisk-e-coli-infektion-ehec/. (Accessed: 21st May 2019).

[3] Söderström A (2008). A large *Escherichia coli* O157 outbreak in Sweden associated with locally produced lettuce. Foodborne Pathog. Dis..

[4] Widgren S (2015). Longitudinal observational study over 38 months of verotoxigenic *Escherichia coli* O157:H7 status in 126 cattle herds. Prev. Vet. Med..

[5] Eriksson E, Aspan A (2005). Prevalence of verotoxin-producing *Escherichia coli* (VTEC) O157 in Swedish dairy herds. Epidemiol. Infect..

[6] Kistemann T, Zimmer S, Vågsholm I, Andersson Y (2004). GIS-supported investigation of human EHEC and cattle VTEC O157 infections in Sweden: Geographical distribution, spatial variation and possible risk factors. Epidemiol. Infect..

[7] Dean-Nystrom EA, Bosworth BT, Moon HW (1997). Pathogenesis of O157:H7 *Escherichia coli* infection in neonatal calves. Adv. Exp. Med. Biol.

[8] Kolenda, R., Burdukiewicz, M. & Schierack, P. A systematic review and meta-analysis of the epidemiology of pathogenic *Escherichia coli* of calves and the role of calves as reservoirs for human pathogenic *E. coli*. Front. *Cell. Infect. Microbiol*. **5** (2015). 10.3389/fcimb.2015.00023 PMC4357325 25815276

[9] Ferens WA, Hovde CJ (2010). *Escherichia coli* O157:H7: Animal reservoir and sources of human infection. Foodborne Pathog. Dis..

[10] Naylor SW (2003). Lymphoid follicle-dense mucosa at the terminal rectum is the principal site of colonization of enterohemorrhagic *Escherichia coli* O157:H7 in the bovine host. Infect. Immun..

[11] Davis MA (2006). Comparison of cultures from rectoanal-junction mucosal swabs and feces for detection of *Escherichia coli* O157 in dairy heifers. Appl. Environ. Microbiol..

[12] Baines D, Lee B, McAllister T (2008). Heterogeneity in enterohemorrhagic Escherichia coli O157:H7 fecal shedding in cattle is related to *Escherichia coli* O157:H7 colonization of the small and large intestine. Can. J. Microbiol..

[13] Chase-Topping ME, Gally D, Low C, Matthews L, Woolhouse M (2008). Super-shedding and the link between human infection and livestock carriage of *Escherichia coli* O157. Nat. Rev. Microbiol.

[14] Matthews L (2006). Heterogeneous shedding of *Escherichia coli* O157 in cattle and its implications for control. Proc. Natl. Acad. Sci. USA.

[15] Arthur TM (2009). Longitudinal study of *Escherichia coli* O157:H7 in a beef cattle feedlot and role of high-level shedders in hide contamination. Appl. Environ. Microbiol..

[16] Cobbold RN (2007). Rectoanal junction colonization of feedlot cattle by *Escherichia coli* O157:H7 and its association with supershedders and excretion dynamics. Appl. Environ. Microbiol..

[17] Chase-Topping ME (2007). Risk factors for the presence of high-level shedders of *Escherichia coli* O157 on Scottish farms. J. Clin. Microbiol..

[18] Spencer SEF, Besser TE, Cobbold RN, French NP (2015). ‘Super’ or just ‘above average’? Supershedders and the transmission of *Escherichia coli* O157:H7 among feedlot cattle. J. R. Soc. Interface.

[19] Widgren S, Engblom S, Emanuelson U, Lindberg A (2018). Spatio-temporal modelling of verotoxigenic *Escherichia coli* O157 in cattle in Sweden: exploring options for control. Vet. Res..

[20] Matthews L (2006). Super-shedding cattle and the transmission dynamics of *Escherichia coli* O157. Epidemiol. Infect..

[21] Besser TE, Richards BL, Rice DH, Hancock DD (2001). *Escherichia coli* O157: H7 infection of calves: infectious dose. Epidemiol Infect.

[22] Sheng H (2016). Standardized *Escherichia coli* O157: H7 exposure studies in cattle provide evidence that bovine factors do not drive increased summertime colonization. Appl. Environ. Microbiol..

[23] Gyles CL (2011). Relevance in pathogenesis research. Vet. Microbiol..

[24] Williams KJ, Ward MP, Dhungyel OP (2015). Daily variations in *Escherichia coli* O157 shedding patterns in a cohort of dairy heifers at pasture. Epidemiol. Infect..

[25] Mechie SC, Chapman PA, Siddons CA (1997). A fifteen month study of *Escherichia coli* O157:H7 in a dairy herd. Epidemiol. Infect..

[26] Smith RP, Pollitt WJ, Paiba GA (2016). A longitudinal study of risk factors for shedding of VTEC O157 by young cattle in herds with known *E. coli* O157 carriage. Epidemiol. Infect..

[27] Robinson SE, Wright EJ, Hart CA, Bennett M, French NP (2004). Intermittent and persistent shedding of *Escherichia coli* O157 in cohorts of naturally infected calves. J. Appl. Microbiol..

[28] Munns KD (2015). Perspectives on super-shedding of *Escherichia coli* O157:H7 by cattle. Foodborne Pathog. Dis..

[29] Wiepkema PR, van Hellemond KK, Roessingh P, Romberg H (1987). Behaviour and Abomasal Damage in Individual Veal Calves. Appl. Anim. Behav. Sci..

[30] Lecorps B, Kappel S, Weary DM, Keyserlingk MAG (2018). Dairy calves’ personality traits predict social proximity and response to an emotional challenge. Sci. Rep..

[31] Fischer-Tenhagen C, Ladwig-Wiegard M, Heuwieser W, Thöne-Reineke C (2018). Short communication: Is hair cortisol a potential indicator for stress caused by chronic lameness in dairy cows?. J. Dairy Sci..

[32] Eitam H, Vaya J, Brosh A, Orlov A (2010). Differential stress responses among newly received calves: variations in reductant capacity and Hsp gene expression. Cell Stress Chaperones.

[33] Lecorps B, Weary DM, Von Keyserlingk MAG (2018). Pessimism and fearfulness in dairy calves. Sci. Rep..

[34] Broom DM (1991). Animal welfare: concepts and measurement. J. Anim. Sci.

[35] Duncan IJH (2005). Science-based assessment of animal welfare: farm animals. Rev. Sci. Tech. – Off. Int. des Epizoot.

[36] Whay HR, Main DCJ, Green LE, Webster AJF (2003). An animal-based welfare assessment of group-housed calves on UK dairy farms. Animal Welfare.

[37] Tamminen L-M (2019). Risk factors and dynamics of verotoxigenic *Escherichia coli* O157:H7 on cattle farms: An observational study combining information from questionnaires, spatial data and molecular analyses. Prev. Vet. Med..

[38] Cray WC, Casey T (1998). Effect of dietary stress on fecal shedding of *Escherichia coli* O157: H7 in Calves. Appl. Environ. Microbiol.

[39] Williams KJ, Ward MP, Dhungyel OP, Hall EJS (2015). Risk factors for *Escherichia coli* O157 shedding and super-shedding by dairy heifers at pasture. Epidemiol. Infect..

[40] Robertson C, Boyle P, Hsieh CC, Macfarlane GJ, Maisonneuve P (1994). Some statistical considerations in the analysis of case-control studies when the exposure variables are continuous measurements. Epidemiology.

[41] Mir RA (2016). Colonization of Beef Cattle by Shiga Toxin-Producing *Escherichia coli* during the First Year of Life: A Cohort Study. PLoS One.

[42] Jeon SJ, Elzo M, DiLorenzo N, Lamb GC, Jeong KC (2013). Evaluation of Animal Genetic and Physiological Factors That Affect the Prevalence of *Escherichia coli* O157 in Cattle. PLoS One.

[43] Veissier I, Boissy A (2007). Stress and welfare: Two complementary concepts that are intrinsically related to the animal’s point of view. Physiol. Behav..

[44] Boyle, L. A. & O’Driscoll, K. Animal welfare: An essential component in food safety and quality *in Food Chain Integrity: A Holistic Approach to Food Traceability, Safety, Quality and Authenticity* (eds Hoorfar, J., Jordan, K., Butler, F., Prugger, R.) 169–186 (Woodhead Publishing, 2011).

[45] Mills, D. S. & Marchant-Forde, J. N. The Encyclopedia of applied animal behaviour and welfare. 115–116 (CABI, 2010).

[46] Spruijt BM, van Hoof JARAM, Gispen WH (1992). Ethology and neurobiology of grooming behavior. Physiol. Rev..

[47] Robinson SE, Brown PE, Wright EJ, Hart CA, French NP (2009). Quantifying within- and between-animal variation and uncertainty associated with counts of *Escherichia coli* O157 occurring in naturally infected cattle faeces. J. R. Soc. Interface.

[48] Perelle S, Dilasser F, Grout J, Fach P (2004). Detection by 5′-nuclease PCR of Shiga-toxin producing *Escherichia coli* O26, O55, O91, O103, O111, O113, O145 and O157:H7, associated with the world’s most frequent clinical cases. Mol. Cell. Probes.

[49] Nielsen EM, Andersen MT (2003). Detection and characterization of verocytotoxin-producing *Escherichia coli* by automated 5′ nuclease PCR assay. J. Clin. Microbiol..

[50] Söderlund R (2014). Molecular typing of *Escherichia coli* O157:H7 isolates from Swedish cattle and human cases: population dynamics and virulence. J. Clin. Microbiol..

[51] Bokkers EAM, Koene P (2001). Activity, oral behaviour and slaughter data as welfare indicators in veal calves: A comparison of three housing systems. Appl. Anim. Behav. Sci..

[52] R Core Team. R: A Language and Environment for Statistical Computing. (2018). https://www.r-project.org/

[53] Wickham, H. ggplot2: Elegant Graphics for Data Analysis. (Springer-Verlag, 2016).

[54] Adobe Inc. Adobe Illustrator. (2017). https://adobe.com/products/illustrator

[55] Stekhoven DJ, Bühlmann P (2012). Missforest-Non-parametric missing value imputation for mixed-type data. Bioinformatics.

[56] Stekhoven, D. J. missForest: Nonparametric missing value imputation using random forest. R package version 1.4. (2013).

[57] Hummel, M., Edelmann, D. & Kopp-Schneider, A. CluMix R package version 2.3.1. (2019).

[58] Hummel, M., Edelmann, D. & Kopp-Schneider, A. Clustering of samples and variables with mixed-type data. *PLoS One* **12** (2017). 10.1371/journal.pone.0188274 PMC5705083 29182671

[59] Friedman J, Hastie T, Tibshirani R (2010). Regularization paths for generalized linear models via Coordinate Descent. J. Stat. Softw..

[60] James, G., Witten, D., Hastie, T. & Tibshirani, R. An Introduction to Statistical Learning: with Applications in R. (Springer Publishing Company, 2013).

[61] Hastie T, Qian J (2014). Glmnet Vignette..

[62] Chavent, M., Kuentz, V., Labenne, A., Benoit, L. & Saracco, J. PCAmixdata: Multivariate Analysis of Mixed Data. (2017).

[63] Bates D, Mächler M, Bolker B, Walker S (2015). Fitting linear mixed-effects models using lme4. J. Stat. Softw..

[64] Rencher, A. C. & Christensen, W. F. Principal component analysis in *Methods of Multivariate Analysis*. 405–434 (Wiley, 2012).

