# Supplementary material for: Socially engaged calves are more likely to be colonised by VTEC O157:H7 than individuals showing signs of poor welfare
Source: Sci Rep. 2020 Apr 14;10:6320. doi: 10.1038/s41598-020-63186-2 (PMC7156447; doi:10.1038/s41598-020-63186-2)
Supplement: Supplementary file 1 — Supplementary information. [file 41598_2020_63186_MOESM1_ESM.pdf]

Supplementary material for:

Socially engaged calves are more likely to be colonised by VTEC O157:H7 than individuals showing signs of poor welfare

L.M. Tamminen[a*], C.R. Hranac[b], J. Dicksved[c], E. Eriksson[d], U. Emanuelson[a], L.J. Keeling[e]

[a] Department of Clinical Sciences, Swedish University of Agricultural Sciences, Box 7054, SE-75007, Uppsala, Sweden

[b] Molecular Epidemiology and Public Health Laboratory (mEpilab), Infectious Disease Research Centre, Hopkirk Research Institute, Massey University, Private Bag 11-222, Palmerston North, New Zealand

[c]Department of Animal Nutrition and Management, Swedish University of Agricultural Sciences, Box 7024, SE-75007, Uppsala, Sweden

[d] National Veterinary Institute (SVA), SE-75189, Uppsala, Sweden

[e] Department of Animal Environment and Health, Swedish University of Agricultural Sciences, Box 7068, SE-75007, Uppsala, Sweden

[*] Corresponding author: Lena-Mari Tamminen, Department of Clinical Sciences, Swedish University of Agricultural Sciences, Uppsala, Sweden, SE-75007

Phone: +4618671428, E-mail: lena.mari.tamminen@slu.se

Supplementary Table S1. Description of indicators used for welfare assessment of the calves.

Individual assessment:	Definition:	Based on:
Body Condition Score	Palpation and scoring from 1 (very thin) to 5 (fat) based on the amount of muscle/fat covering over the lumbar vertebrae's and hips bones. Split into Poor (score 1-2) and Normal (3-4). No score 5 was observed.	1
Cleanliness:		
Below hocks<br>Upper hind legs<br>Body	Ocular observation and scoring from 1 to 3: No dirt; Minor splashing of manure (smaller than a hand in total); Distinct dried plaques of manure (larger than a hand) or 50% of area covered in wet manure	2–4
Coat condition	Ocular observation and scoring : Poor (dull, shaggy or long rough hairs); Normal (shiny and short hairs)	3
Conjunctivitis	Ocular observation of inflammation (redness/swelling) around the eye	
Fearfulness (Distance Avoidance test)	Freestanding calf is approached with a speed of 1 step per second with experimenter aiming at reaching shoulder level. Stop at a distance of one arm's length and arm is lifted to touch the animal. Distance when avoidance reaction occurs noted. Scores 1 to 5: >2 m, <2m, <1m, withdrawal when arm is lifted or if the animal can be touched (for at least 2s) were collapsed to scores 1 to 3: >2 m, <2m, <1m.	5
Faecal consistency	Ocular observation and assessment of consistency. Scored as: Diarrhoea (faeces watery or slimy); Loose (faeces smooth and homogenous); Firm (faeces firm and break up)	
Hairless patches	Ocular observation of number of areas >2 cm^2 with hair loss, extensive thinning of the coat due to parasites or hyperkeratosis with non-damaged skin were calculated. Up to 20 areas were counted. One area larger than the size of a hand counted as 20 small areas.	3
Lameness	Ocular observation and scoring from 1-3: No lameness; Uneven temporal rhythm; Reluctance to bear weight on a leg	
Nasal discharge	Ocular observation of flow/discharge from the nostrils	3
Number of wounds/inflammation	As described above for Hairless patches but observation and counting of areas with damaged skin either in form of a scab or a wound, dermatitis due to ectoparasites or ear lesions due to torn off ear tags	3
Reactivity (during sampling)	Assessed during restraining for hair-sampling. Animal scored from 1-4 after release: 1. Animal is relaxed and not trying to resist being restrained. Curious and/or contact-seeking toward persons performing the sampling; 2. Animal is nervous and stepping around during sampling. May try to avoid sampling by moving the hind and lifting hind legs although not kicking; 3. Animal is forcefully trying to avoid being restrained by trying to escape or forcefully kicks; 4. Animal is paralyzed by the restraining. Increased respiration rate and introvert behaviour. No forceful attempts to escape or react to the sampling	
Ruminal fill*	Ocular observation and scoring as: Poor (visible triangle formed in left para lumbar fossa and small rumen[1]); Normal (filled rumen is visible in Para lumbar fossa)	6
Size within group	Ocular observation of group and scoring as: Small (calf is smaller than the average size in the group); Normal (calf size does not differ from other animals); Large (calf is larger than the average size in the group)	
Tearing (epiphora)	Ocular observation of discharge from the eyes. Estimated length of tearstain noted	
**Behavioural observation†**		
Butting	Animal pushes front head against other calf but receiving animal does not move	7
Coughing	Expelling air from the lungs with a sudden sharp sound.	
Cross-sucking	Calf performs sucking movements when having part of another calf's body in its mouth. Could also include sucking at prepuce or testis	8
Displacement	Animal displaces another calf by pushing forehead or base of head against other calf and thereby forcing it to move	7
Fighting	Two calves butting heads standing front to front.	9
Headshake	Shaking or rotating head	9
Licking other calf	Any part of other calf is licked one or multiple times without pause	7

Locomotor play	Running, turning, jumping, bucking or play with environment (for example playful butting of objects/pen)	9
Mounting	Calf mounts another calf from any direction	9
Oral manipulation of environment (e.g. pen, floor)	Licking, nibbling or suckling objects or environment	7
Rubbing/scratching	Repeatedly rubbing claw of hind leg over head, neck shoulder or moving body against objects/wall/other calf	7,10
Self-licking	Licking any part of own body	10
Stretching	Stretching back, tail, hind legs or neck separately or simultaneously	10
Tongue playing/rolling	Swaying, turning and partly rolling and unrolling the tongue inside the open mouth or when tongue is extended	7

**Calves<2 weeks of age given milk – all assessed as normal.*

†*Frequency of behaviours observed during 20 minutes per pen.*

References:

1. VÄXA Sverige. *Fråga Kon.*
2. Leach, K. A., Knierim, U. & Whay, H. R. Cleanliness scoring for dairy and beef cattle and veal calves. in *Animal Welfare Measures of Dairy Cattle, Beef Bulls and Veal Calves - Welfare Quality Reports No. 11* (eds. Keeling, L. J. & Forkman, B.) 25–30 (2009).
3. Welfare Quality®. Welfare Quality® assessment protocol for cattle. in *CONSORTIUM, W. Q. (ed.)* (2009).
4. Tamminen, L.-M. An investigation of the association between animal welfare and shedding of Cryptosporidium in calves from two dairy farms. **59**, (Swedish University of Agricultural Sciences, 2013).
5. Windschnurer, I., Schmied, C., Boivin, X. & Waiblinger, S. Reliability and inter-test relationship of tests for on-farm assessment of dairy cows' relationship to humans. *Appl. Anim. Behav. Sci.* **114**, 37–53 (2008).
6. Whay, H. R., Main, D. C. J., Green, L. E. & Webster, A. J. F. An animal-based welfare assessment of group-housed calves on UK dairy farms. *Animal Welfare* **12**, 611–617 (2003).
7. Bokkers, E. A. M. *et al.* Inter-observer and test-retest reliability of on-farm behavioural observations in veal calves. in *Animal Welfare* **18**, 381–390 (2009).
8. Lidfors, L. M. Cross-sucking in group-housed dairy calves before and after weaning off milk. *Appl. Anim. Behav. Sci.* **38**, 15–24 (1993).
9. Jensen, M. B. & Kyhn, R. Play behaviour in group-housed dairy calves, the effect of space allowance. *Appl. Anim. Behav. Sci.* **67**, 35–46 (2000).
10. Bokkers, E. A. M. & Koene, P. Activity, oral behaviour and slaughter data as welfare indicators in veal calves: A comparison of three housing systems. *Appl. Anim. Behav. Sci.* **75**, 1–15 (2001).

Supplementary Table S2. Descriptive statistics and univariable analysis of variables included in the analysis. Quantitative variables are presented in italics with average in group and range (minimum and maximum). Univariable analysis shows results from a logistic regression model with colonisation status (as detected by rectoanal mucosal swabs) as response variable. The presented variable and pen (as random effect to account for clustering) were included as explanatory variables. Logistic regression was performed in R using the package lme4.

		Status VTEC O157:H7		Univariable analysis	
		Non-colonised *n=135*	Colonised *n=56*	OR (95% conf int)	p-value
Age (days)	*Average (min – max)*	*138.8 (19-302)*	*113.4 (19-255)*	0.993 (0.99-1.00)	0.037
Sex	Female	88	47		0.037
	Male	30	7	0.43 (0.15-1.09)	
	Castrated male	17	2	0.18 (0.03-0.79)	
	Missing	0	0		
Size	Normal	82	40		0.28
	Small	29	6	0.44 (0.15-1.17)	
	Large	23	10	0.92 (0.36-2.23)	
	Missing	1	0		
BCS	Normal (3-5)	103	47		0.21
	Poor (1-2)	31	8	0.55 (0.21-1.34)	
	Missing	1	1		
Cleanliness below hocks	1	31	19		0.11
	2	86	33	0.57 (0.24-1.27)	
	3	15	2	0.17 (0.02-0.82)	
	Missing	3	2		
Cleanliness body	1	55	29		0.31
	2	61	22	0.67 (0.33-1.35)	
	3	14	3	0.40 (0.08-1.46)	
	Missing	5	2		
Cleanliness upper hind	1	23	14		0.36
	2	73	28	0.58 (0.24-1.37)	
	3	36	12	0.50 (0.18-1.38)	
	Missing	3	2		
Coat condition	Normal	82	38		0.35
	Abnormal	50	17	0.71 (0.33-1.44)	
	Missing	3	1		
Hairless spots	None	76	36		0.44
	Few	43	14	0.62 (0.28-1.33)	

	Many	14	5	0.62 (0.16-1.99)	
	Missing	2	1		
Inflammation (wounds/swelling)	No	115	51		0.18
	Yes	18	4	0.42 (0.10-1.36)	
	Missing	2	1		
Tearing (epiphora)	0-2 cm	82	35		0.66
	2-5 cm	33	14	0.99 (0.44-2.18)	
	> 5 cm	19	6	0.60 (0.17-1.73)	
	Missing	1	1		
Nasal discharge	No	83	30		0.41
	Yes	50	25	1.35 (0.66-2.73)	
	Missing	2	1		
Fecal consistency	Loose	81	37		0.09
	Firm	34	17	1.03 (0.47-2.16)	
	Abnormal (diarrhoea)	20	1	0.1 (0.01-0.54)	
	Missing	0	1		
Distance avoidance test	1-2m	44	18		0.62
	> 2m	36	13	0.81 (0.32-1.99)	
	< 1m (incl. touch and withdrawal when arm is lifted)	37	13	0.81 (0.32-1.97)	
	Missing by design	10	8	1.85 (0.48-6.91)	
	Missing (at random)	8	4		
Reactivity	Low	60	24		0.37
	Average	49	25	1.31 (0.64-2.70)	
	High	26	6	0.63 (0.21-1.77)	
	Missing	0	1		
Cough	No	97	39		0.99
	Yes	37	16	1.00 (0.46-2.07)	
	Missing	1	1		
	Average frequency of behaviour/calf (min – max)	*0.42 (0-5)*	*0.83 (0-8)*	*1.28 (1.00-1.67)*	*0.06*
Butting other	No	124	47		0.31
	Yes	11	9	1.72 (0.57-4.83)	
	Average frequency of behaviour/calf (min – max)	*0.1 (0-2)*	*0.2 (0-3)*	*1.45 (0.63-3.32)*	*0.37*
Butted by other	No	125	48		0.29

	Yes	10	8	1.78 (0.58-5.14)	
	Average frequency of behaviour/calf (min – max)	*0.10 (0-2)*	*0.20 (0-2)*	*1.52 (0.70-3.25)*	*0.27*
Cross-sucking other	No	120	53		0.21
	Yes	15	3	0.42 (0.09-1.48)	
	Average frequency of behaviour/calf (min – max)	*0.26 (0-8)*	*0.16 (0-4)*	*0.85 (0.50-1.23)*	*0.45*
Cross-sucked by other	No	126	51		0.47
	Yes	9	5	1.58 (0.43-5.38)	
	Average frequency of behaviour/calf (min – max)	*0.17 (0-10)*	*0.16 (0-8)*	*0.97 (0.60-1.40)*	*0.87*
Displacing	No	125	51		0.75
	Yes	10	5	1.22 (0.34-3.93)	
	Average frequency of behaviour/calf (min – max)	*0.08 (0-2)*	*0.29 (0-6)*	*1.57 (0.94-3.25)*	*0.13*
Displaced	No	124	52		0.79
	Yes	11	4	0.84 (0.21-2.86)	
	Average frequency of behaviour/calf (min – max)	*0.09 (0-2)*	*0.16 (0-5)*	*1.32 (0.66-2.85)*	*0..42*
Fighting	No	128	55		0.25
	Yes	7	1	0.27 (0.01-1.80)	
	Average frequency of behaviour/calf (min – max)	*0.06 (0-2)*	*0.02 (0-1)*	*0.30 (0.02-1.48)*	*0.23*
Headshake	No	130	55		0.30
	Yes	5	1	0.29 (0.01-2.19)	
	Average frequency of behaviour/calf (min – max)	*0.05 (0-2)*	*0.09 (0-5)*	*1.06 (0.46-2.40)*	*0.87*
Licking other	No	112	42		0.22
	Yes	23	14	1.67 (0.72-3.83)	
	Average frequency of behaviour/calf (min – max)	*0.47 (0-7)*	*0.30 (0-3)*	*0.86 (0.58-1.17)*	*0.39*
Licked by other	No	100	40		0.65
	Yes	35	16	1.19 (0.55-2.57)	
	Average frequency of behaviour/calf (min – max)	0.47 (0-4)	0.46 (0-5)	*1.03 (0.70-1.48)*	*0.86*

Locomotor play	No	125	51		0.69
	Yes	10	5	1.29 (0.35-4.39)	
	Average frequency of behaviour/calf (min – max)	*0.10 (0-2)*	*0.18 (0-4)*	*1.39 (0.70-2.76)*	*0.33*
Mounting	No	130	56		
	Yes	5	0	Estimation not possible	
	Average frequency of behaviour/calf (min – max)	*0.04 (0-1)*	*0 (0-0)*	*Estimation not possible*	
Mounted by other		128	56	Estimation not possible	
		7	0		
	Average frequency of behaviour/calf (min – max)	*0.05 (0-1)*	*0 (0-0)*	*Estimation not possible*	
Oral manipulation pen	No	99	36		0.30
	Yes	36	20	1.46 (0.70-2.97)	
	Average frequency of behaviour/calf (min – max)	*0.55 (0-6)*	*0.77 (0-6)*	*1.15 (0.88-1.49)*	*0.31*
Self-rubbing	No	97	40		0.97
	Yes	38	16	0.99 (0.45-2.09)	
	Average frequency of behaviour/calf (min – max)	*0.44 (0-7)*	*0.45 (0-4)*	*0.99 (0.66-1.43)*	*0.98*
Rubbed by other	No	120	50		0.90
	Yes	15	6	0.94 (0.30-2.64)	
	Average frequency of behaviour/calf (min – max)	*0.13 (0-2)*	*0.11 (0-1)*	*0.84 (0.29-2.10)*	*0.72*
Rubbing on other	No	123	54		0.20
	Yes	12	2	0.35 (0.05-1.46)	
	Average frequency of behaviour/calf (min – max)	*0.10 (0-2)*	*0.04 (0-1)*	*0.36 (0.05-1.35)*	*0.19*
Self-licking	No	50	10		0.01
	Yes	85	46	2.91 (1.33-6.93)	
	Average frequency of behaviour/calf (min – max)	*1.94 (0-11)*	*2.14 (0-8)*	*1.04 (0.90-1.19)*	*0.58*
Stretching	No	130	55		0.46
	Yes	5	1	0.42 (0.02-3.25)	
	Average frequency of behaviour/calf (min – max)	*0.04 (0-1)*	*0.02 (0-1)*	*0.42 (0.02-3.25)*	*0.46*

Tongue playing/rolling	No	127	49		0.11
	Yes	8	7	2.65 (0.79-9.07)	
	Average frequency of behaviour/calf	*0.10*	*0.20*	*1.50 (0.78-2.87)*	*0.21*

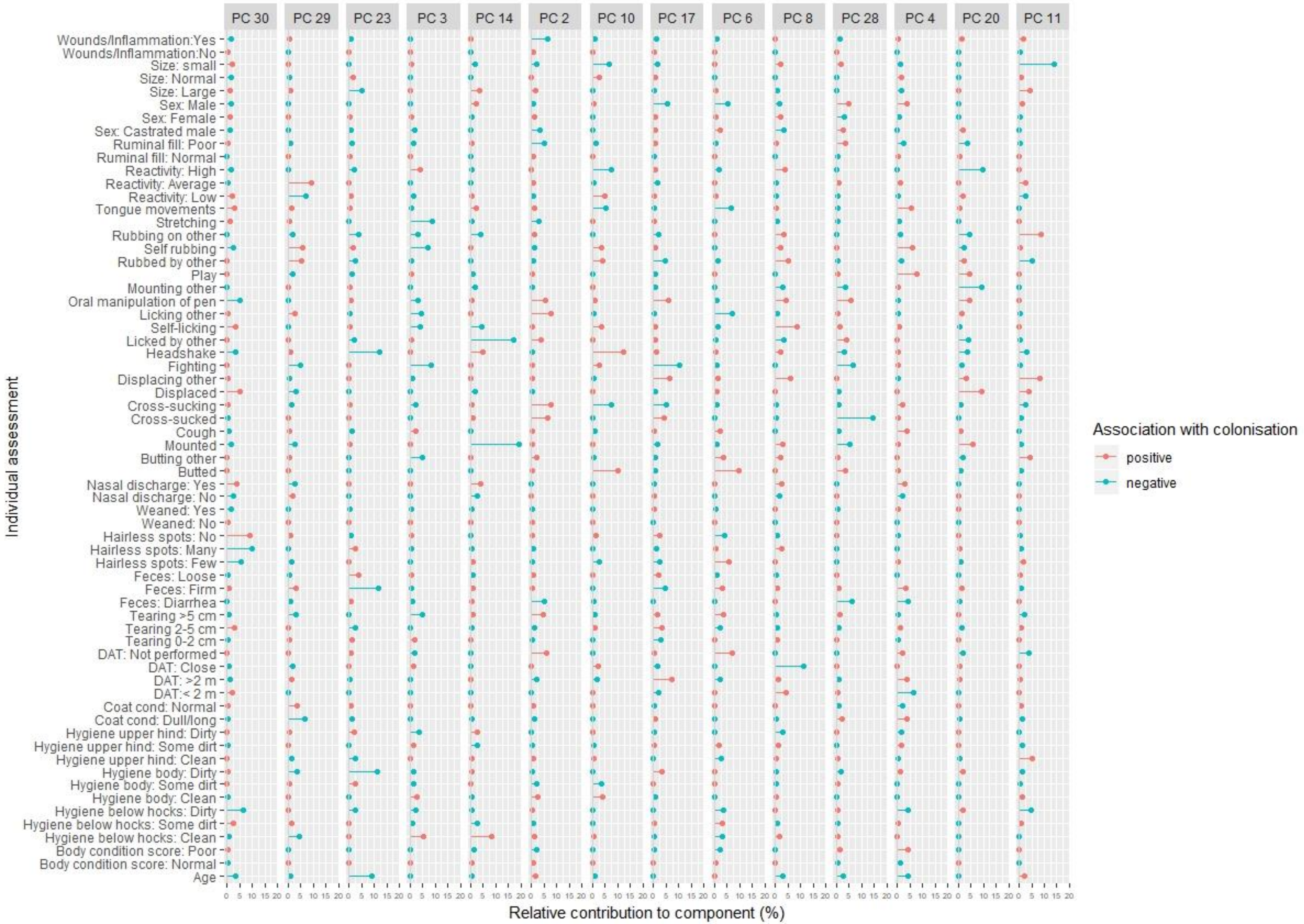

Supplementary figure S3. Variable contributions to Principal Components associated with colonisation of VTEC O157:H7 in the Principal component regression model.

Supplementary Table S4. Description and result from sampling of pens indicated as positive for VTEC O157:H7 by environmental sampling.

Farm	Date of sampling	Pen	Animals in pen	Sampled animals	Colonised animals	Shedding animals	Pen size (m2)	Bedding	Average age (days)	Comments
**F1**	2015-10-26	1	5	4	4	1	11	Straw	94	Sampling of last calf not performed as it jumped out of the pen
		2	8	5	2	0	11	Straw	106	
		3	6	6	0	0	6	Slatted floor/rubber	149	
		4	6	6	0	0	6	Slatted floor/rubber	227	
		5	6	3	0	0	13	Straw	127	Sampling stopped after 3 animals due to stress in the group
**F2**	2015-11-16	1	11	8	1	0	32	Straw	145	
		2	12	5	1	1	36	Straw	196	
		3	10	6	0	0	29	Straw	75	
		4	11	7	0	0	30	Straw	111	
**F3**	2015-10-20	1	20	10	1	1	59	Straw	85	
		2	10	1	0	0	79	Straw	155	Sampling stopped because of risk of injury
		3	9	4	0	0	34	Straw	101	
		4	16	5	0	0	78	Straw	148	
**F4**	2015-11-24	1	7	7	2	1	27	Straw	111	
		2	13	13	0	0	84	Straw	158	
**F5**	2015-11-17	1	14	9	3	1	65	Straw	154	
		2	14	9	0	0	65	Straw	137	
		3	7	5	0	0	31	Straw	97	
		4	8	5	0	0	31	Straw	82	
**F6**	2016-11-02	1	4	3	1	1	6	Straw	51	
		2	20	14	10	0	48	Straw	99	
		3	4	3	0	0	6	Straw	50	
		4	4	3	0	0	6	Straw	33	
		5	4	3	0	0	6	Straw	34	
**F7-1**	2015-11-18	1	6	6	2	0	13	Straw	49	
		2	4	4	3	1	13	Straw	95	
		3	6	4	0	0	17	Straw	12	Two calves that were <48 hours old were not sampled
		4	17	3	0	0	60	Straw	127	Sampling stopped after 3 animals because risk of injuries (No adequate possibilities for restraining)
**F7-2**	2016-11-03	1	7	7	3	1	13	Straw	78	

		2	7	7	1	1	13	Straw	121	
		3	1	1	0	0	13	Straw	42	
		5	5	5	0	0	17	Straw	30	
**F8**	2016-04-28	1	18	8	1	1	150	Straw	219	
		2	8	5	1	0	26	Straw	175	
		3	12	8	1	0	36	Straw	118	
		4	7	4	0	0	21	Straw	46	
**F9-1**	2015-10-27	1	36	14	1	1	190	Slatted floor/rubber	176	
		2	8	5	2	2	20	Straw	98	
		3	10	5	2	1	22	Straw	101	
		4	11	5	0	0	22	Straw	76	
**F9-2**	2016-06-03	5	8	7	4	2	22	Straw	101	
		2	6	6	0	0	20	Straw	121	
		3	7	7	0	0	22	Straw	139	
**F10**	2016-06-28	1	12	12	2	2	25	Straw	61	
		2	13	7	1	0	128	Straw	195	Poor restraining possibilities led to time running out
		3	6	6	0	0	30	Sawdust	134	
**F11**	2016-04-06	1	4	4	3	0	4	Straw	71	
		2	6	6	2	0	8	Slatted floor/rubber	171	
		3	3	3	1	0	3	Straw	43	
		4	3	2	0	0	6	Straw	143	
		6	3	3	0	0	5	Straw	135	
		7	2	2	0	0	2	Straw	12	
**F12**	2017-04-17	1	19	18	3	2	137	Straw	216	Unsampled animal an older cow
